# Genome Analysis of the Broad Host Range Necrotroph *Nalanthamala psidii* Highlights Genes Associated With Virulence

**DOI:** 10.3389/fpls.2022.811152

**Published:** 2022-02-25

**Authors:** Anita A. Severn-Ellis, Maritha H. Schoeman, Philipp E. Bayer, James K. Hane, D. Jasper G. Rees, David Edwards, Jacqueline Batley

**Affiliations:** ^1^School of Biological Sciences, Institute of Agriculture, The University of Western Australia, Crawley, WA, Australia; ^2^Aquaculture Research and Development, Department of Primary Industries and Regional Development, Indian Ocean Marine Research Centre, Watermans Bay, WA, Australia; ^3^Institute for Tropical and Subtropical Crops, Agricultural Research Council, Nelspruit, South Africa; ^4^Centre for Crop and Disease Management, School of Molecular and Life Sciences, Curtin University, Bentley, WA, Australia; ^5^Agricultural Research Council, Biotechnology Platform, Pretoria, South Africa; ^6^Botswana University of Agriculture and Natural Resources, Gaborone, Botswana

**Keywords:** *Nalanthamala psidii*, necrotroph, secretome, effectors, secondary metabolites, regulatory proteins, transporters, guava wilt disease

## Abstract

Guava wilt disease is caused by the fungus *Nalanthamala psidii*. The wilt disease results in large-scale destruction of orchards in South Africa, Taiwan, and several Southeast Asian countries. *De novo* assembly, annotation, and in-depth analysis of the *N. psidii* genome were carried out to facilitate the identification of characteristics associated with pathogenicity and pathogen evolution. The predicted secretome revealed a range of CAZymes, proteases, lipases and peroxidases associated with plant cell wall degradation, nutrient acquisition, and disease development. Further analysis of the *N. psidii* carbohydrate-active enzyme profile exposed the broad-spectrum necrotrophic lifestyle of the pathogen, which was corroborated by the identification of putative effectors and secondary metabolites with the potential to induce tissue necrosis and cell surface-dependent immune responses. Putative regulatory proteins including transcription factors and kinases were identified in addition to transporters potentially involved in the secretion of secondary metabolites. Transporters identified included important ABC and MFS transporters involved in the efflux of fungicides. Analysis of the repetitive landscape and the detection of mechanisms linked to reproduction such as *het* and mating genes rendered insights into the biological complexity and evolutionary potential of *N. psidii* as guava pathogen. Hence, the assembly and annotation of the *N. psidii* genome provided a valuable platform to explore the pathogenic potential and necrotrophic lifestyle of the guava wilt pathogen.

## Introduction

Guava (*Psidium guajava* L.), a tropical fruit crop belonging to the family Myrtaceae (Nakasone and Paull, 1998) gained commercial significance due to its versatility and nutritional value. Guava wilt disease (GWD), caused by *Nalanthamala psidii*, was first reported in Taiwan in 1923 (Kurosowa, 1926). The occurrence of GWD in Taiwan increased to epidemic proportions in the 70’s and resulted in large scale destruction of orchards (Wan and Leu, 1999). Outbreaks of GWD have also been reported in several South-East Asian countries, including Thailand (Athipunyakom, 2008), Malaysia (Schoeman, 1997), and the Philippines (Quimio et al., 1984; Opina, 1995). More recently, severe losses in production were reported in Bangladesh as a result of guava wilt disease (Alam et al., 2019).

Infected trees gradually decline, with leaves initially turning yellow and changing over time to red and brown. Leaf drop occurs gradually, eventually resulting in the complete defoliation of the tree (Schoeman et al., 1997). In some instances, a rapid sectorial decline of the tree is observed whereby leaves on affected branches wilt and die, and fruit development ceases. Immature fruits are abscised while more mature fruits remain attached and mummify on the tree (Schoeman et al., 1997; Grech, 2015). Wood discolouration usually occurs during the advanced stages of the disease. Development of gray to reddish-brown blisters on the trunks and branches follows from which white to salmon colored conidial masses and sporodochia are subsequently released (Schoeman et al., 1997). Sporodochia can also be found on exposed roots.

*Nalanthamala psidii* was first described as *Myxosporium psidii* (Sawada and Kurosawa) by Kurosawa in 1926 (Kurosowa, 1926). The classification was based on acervuli-like conidiomata, penicillate and simple branched conidiophores, as well as two distinct types of conidia (Schroers et al., 2005). The fungus has also been described as *Septofusidium* sp. (Grech, 2015), *Acremonium diospyri* (Benade et al., 1991) and *Penicillium vermoesenii* (Schoeman et al., 1997). The re-classification of *M. psidii* as *N. psidii* has been suggested based on the analyses of the internal transcribed spacer regions and 5.8S rDNA (ITS rDNA), LSU rDNA, and partial β-tubulin gene. Polymorphic sites in the ITS rDNA and β-tubulin gene further indicated that *N. psidii* consisted of two lineages assigned to Malaysia/Taiwan and South Africa, respectively (Schroers et al., 2005).

In South Africa, the outbreak of guava wilt disease in the 1980s resulted in devastating losses of more than half of the guava industry in the northern and eastern regions of the country (Schoeman et al., 1997). Affected areas were replanted with a resistant guava variety (‘TS-G2’) developed by the Agricultural Research Council’s Institute for Tropical and Subtropical Crops (Vos et al., 1998). However, plant resistance was soon overcome in boom-and-bust style (Stakman, 1957) by the pathogen, threatening the existence of the guava industry once again. Many farmers have since had no choice but to turn to alternative crops leading to the subsequent closure of processing plants and job losses in affected areas.

Chemical control measures have proven ineffective in the management of GWD (Schoeman et al., 2017). The use of resistant varieties would therefore pose a more effective strategy in the control of GWD. However, breeding for resistance in tree crops, such as guava, is both challenging and time-consuming. The emergence of additional GWD pathogen races poses a further obstacle in the development of varieties with durable resistance. Fungal phytopathogens are characterized by remarkable genetic flexibility that facilitates rapid evolution and adaptation to their host and environment (Calo et al., 2013). According to Howlett et al. (2015), the evolutionary risk or potential of fungal pathogens to evolve virulence is governed by features of their biology and genome. Elucidating the characteristics associated with pathogenicity and pathogen evolution is therefore crucial in the development of effective disease management strategies (McDonald and Linde, 2002).

Advances in technologies and reduction in cost have encouraged the sequencing of an increasing number of fungal genomes, including numerous plant-pathogenic fungi (Grigoriev et al., 2014; Aylward et al., 2017). The pace in genomic growth was met with analogous progress in supporting bioinformatics and the development of numerous reference databases (Edwards and Batley, 2010; Hu et al., 2018). The resulting resources have created an enabling framework for genome characterization and accelerated the discovery of disease-related genes (Howlett et al., 2015; Aylward et al., 2017).

Here, we report on the *de novo* assembly of the *N. psidii* genome, the first to be sequenced within the *Nalanthamala* genus. The genomic resource provided a platform to explore the pathogenic potential of the GWD pathogen. Systematic analysis of the putative protein-coding component of the genome was carried out to identify secreted enzymes and effectors involved in host colonization and the acquisition of nutrients as well as biosynthetic pathways for secondary metabolites, including toxins that can cause tissue necrosis. Additionally, gene regulators and promotors of pathogen adaptation such as peroxidases, Cytochrome P450, transcription factors, kinases, as well as cellular transporters involved in fungicide resistance and the secretion of secondary metabolites were identified as well as genes and genomic features involved in fungal evolution. The study provides insights into the genome and lifestyle of *N. psidii* and context for the development of effective disease management strategies for GWD.

## Materials and Methods

### *N. psidii* Isolate, DNA and RNA Extraction

The *N. psidii* strain MA3 (PPRI 10309) used in the study was isolated from the guava variety ‘Fan Retief’ and maintained by M. Schoeman from the ARC Institute of Tropical and Subtropical Crops in Nelspruit, South Africa.

A single spore culture was grown on Malt Extract Agar overlaid with a single layer of cellulose membrane (Cassago et al., 2002) for 5 days at 25°C. Genomic DNA was extracted from the mycelial mats using the Qiagen DNeasy Plant kit (QIAGEN Co., Ltd., Hamburg, Germany) according to the manufacturer’s instructions. RNA for transcriptome sequencing was extracted from germinating spores and mycelial mats collected, respectively, after 2 and 7 days of incubation using the RNeasy Plant Mini kit (QIAGEN Co., Ltd., Hamburg, Germany) according to the manufacturer’s protocol for total RNA from plant cells and tissues and filamentous fungi.

### Whole-Genome and Transcriptome Sequencing and Assembly

DNA paired-end (PE) libraries with insert sizes of 200, 300, and 500 bp were prepared using the Nextera DNA Library Prep Kit (Illumina), while mate-pair libraries with an insert size of 5 and 9 kb were prepared using the Nextera Mate Pair Library Prep Kit (Illumina Inc., San Diego, CA, United States) (Syed et al., 2009). RNA-Seq libraries were prepared using the TruSeq Stranded Total RNA Kit with Ribo-Zero Plant (Illumina Inc., San Diego, CA, United States) according to the manufacturer’s instructions. Sequencing libraries were prepared by the ARC Biotechnology Platform Core staff (Onderstepoort, South Africa) and sequenced on the Illumina HiScanSQ and MiSeq platforms.

Paired-end reads were trimmed for assembly using Trimmomatic v0.33 (Bolger et al., 2014) to remove sequencing adapters, primers, and low-quality reads. Mate-paired reads for scaffold extension were prepared using Nxtrim (O’Connell et al., 2015) to remove Nextera Mate-pair adapters and categorize reads according to their orientation as implied by their adapter location. Using default settings, four output files were generated. The large insert-sized mate-pair reads (mp files) were then passed through Trimmomatic v0.33 (Bolger et al., 2014) and quality filtered. The remaining reads were merged using FLASH-1.2 (Magoč and Salzberg, 2011) to remove any remaining overlapping reads.

*De novo* assembly was carried out using SPAdes v3.7 (Bankevich et al., 2012), Quast v5.0.2 (Gurevich et al., 2013) was used thereafter to evaluate the genome assembly. Additional scaffolding was carried out with SSPACE 2.0 (Boetzer et al., 2011) using prepared 5 and 9 kb mate paired libraries. Two successive rounds of scaffolding were intermitted with a round of GAPCLOSER2 (Li et al., 2010) using paired-end reads to fill in scaffold gaps. Scaffolds less than 200 bp in size were finally removed. The genome assembly pipeline was based on the pipeline described by Hane et al. (2014). The Benchmarking Universal Single-Copy Orthologs (BUSCO v3.0.2 tool) (Simão et al., 2015) was used to quantify genome completeness using 290 conserved orthologous genes for fungi, while the genome statistics were calculated using QUAST v5.0.2 (Gurevich et al., 2013). The genome of the *N. psidii* MA3 strain (PPRI 10309) is available from the University of Western Australia repository (doi: 10.26182/dbe0-mc39).

The adapter sequences and low-quality reads of RNAseq data were removed using Trimmomatic v0.33 (Bolger et al., 2014). RNA reads were aligned to the genome using the splice junction mapper TopHat2 v2.1.0 (Trapnell et al., 2009; Kim et al., 2013) with default settings. Transcriptome-based gene structures were obtained using Cufflinks v2.1.1 run with default parameters and –max-intron-length set to 5000 bp for fungal assemblies (Trapnell et al., 2012). *De novo* assembly of trimmed *in vitro* expressed RNA-Seq reads was performed using Trinity v2.4.0 (Grabherr et al., 2011) with the default parameters and –jaccard_clip recommended for gene-dense compact fungal genomes. *In silico* normalization was carried out by default with a maximum read coverage of 50.

### Repeat Elements, Non-coding Gene Annotation and Repeat-Induced Point Mutations Analysis

*De novo* and species-specific repeats were predicted using RepeatModeller v1.0.11^1^. The predicted repeat component was amended with the RepBase (2016) (Bao et al., 2015) library to mask assembly scaffolds using RepeatMasker (Tarailo-Graovac and Chen, 2009) before gene prediction. Fungi were selected as species and rmblastn as search engine. Predicted TEs were further classified using the RepBase (Bao et al., 2015) classification system. Transfer RNAs were predicted using tRNAscan-SE 1.3.1 (Lowe and Eddy, 1997).

RIPCAL v2 was used to analyze repeat-induced point mutations (RIP) in genomic DNA repeats by performing RIP indexing and alignment-based analyses (Hane and Oliver, 2008). OcculterCut (Testa et al., 2016) was used to identify AT-rich regions and genes undergoing RIP-assisted diversifying selection, such as small secreted effector proteins involved in the mediation of the host-pathogen interactions.

### Gene Prediction

Gene prediction was carried out similarly to the pipeline described by Bertazzoni et al., 2021. Semi-supervised gene predicting software GeneMark-ET v4.33 (Borodovsky and Lomsadze, 2014) and self-training fungal gene prediction tool CodingQuarry v2 (Testa et al., 2015) was used to predict gene structures based on the mapped transcriptome. CodingQuarry (QC/QCPM) was run with the “run_CQ-PM_stranded.sh” script to allow the prediction of effector-like genes, which might be overlooked in standard predictions. The homology-based Analysis and Annotation Tool AAT (Huang et al., 1997) was used to map the protein coding genes and ESTs of *Beauveria bassiana* ARSEF 286 (Xiao et al., 2012) *Botryosphaeria dothidea* (Marsberg et al., 2017), *Colletotrichum graminicola* M1.001 (O’Connell et al., 2015), *Eutypa lata* UCREL1 (Blanco-Ulate et al., 2013), *Fusarium graminearum* strain PH-1 (NRRL 31084) (Ma et al., 2010) *Fusarium fujikuroi* IMI 58289 (Wiemann et al., 2013), *Fusarium verticillioides* 7600 v2 (Cuomo et al., 2007; Ma et al., 2010), *Grosmannia clavigera* kw1407 (DiGuistini et al., 2011), *Ilyonectria* sp. (Liao et al., 2019), *Nectria haematococca* MPV1, strain 77-13-4 (Coleman et al., 2009), *Ophiostoma piceae* UAMH 11346 (Haridas et al., 2013), *Trichoderma reesei* (Martinez et al., 2008) *Verticillium dahliae* Vdls. 17 (Klosterman et al., 2011) to the *N. psidii* MA3 genome to assist in the location and definition of intron and exon boundaries. The protein-coding genes and EST data from the accessions were obtained from the MycoCosm database (Grigoriev et al., 2014). Transcriptome alignments created with TopHat2 and Cufflinks, as described previously, were used to identify candidate coding regions using TransDecoder v3.0.1^2^. *De novo* transcriptome assembly was first mapped and aligned to the genome with GMAP-GSNAP v2017-06-20 (Wu and Watanabe, 2005) and submitted to PASAlite^3^ to establish exon structures. Finally, weighted consensus gene structure annotations were computed using EvidenceModeler v1.1.1 (EVM) (Haas et al., 2008) based on transcript evidence, semi-supervised and homology-based gene models.

### Functional Annotation of the Protein Content

The predicted protein-coding sequences were functionally annotated using InterProScan v5.26-65.0 (Quevillon et al., 2005) with the Pfam (Punta et al., 2012), PRINTS (Attwood et al., 2012), SMART (Letunic et al., 2011), and SUPERFAMILY (de Lima Morais et al., 2011) databases. The predicted proteome was also submitted to eggNOG-mapper v2 (Huerta-Cepas et al., 2016; Huerta-Cepas et al., 2017), Cluster of Orthologous Groups (COG) (Tatusov et al., 2000) as well as Kyoto Encyclopedia of Genes and Genomes (KEGG) (Kanehisa and Goto, 2000; Kanehisa et al., 2019) to complement the functional annotation created.

### Annotation of Specific Gene Categories

Carbohydrate-active enzyme (CAZymes) annotations were assigned to the predicted proteome from CAZyme family-specific HMM profiles (dbCAN HMMdb v9) from the dbCAN2 database (Cantarel et al., 2009; Yin et al., 2012; Lombard et al., 2014) and HMMER v3.0 hmmscan program. The protease component of the predicted proteome was distinguished using the MEROPS database (Rawlings et al., 2014, 2016) and BLASTP (v2.2.6) (Altschul et al., 1990) with a search threshold E-value of 1e-5. The dedicated fungal peroxidase database fPoxDB (Choi et al., 2014) along with Pfam was used to identify putative genes associated with peroxidases. The RedoxiBase^4^ was used as a guideline to search Pfam results to identify oxidases and other oxidoreductase enzymes. Putative transmembrane transporter proteins were identified through the BLASTP search (threshold *E*-value of 1e-5) of the Transporter Classification Database (TCDB) (Saier et al., 2006, 2016) and verified by comparing InterProScan, Pfam, PRINTS and Superfamily results. Kinomes were identified using Kinannote (Goldberg et al., 2013), while the transcription factor DNA binding domains (TF DBDs in *N. psidii* were identified by interrogating the InterProScan results for TF families and InterProScan terms defined by the Fungal Transcription Factor Database and those described by Shelest (2017). BLASTP v2.2.6 search of the Fungal Cytochrome P450 Database (Park et al., 2008) was conducted to identify predicted Cytochrome P450 enzymes and compared to InterProScan, Pfam, PRINTS and Superfamily results. Genes potentially associated with pathogenicity were identified by aligning the predicted proteome against PHI-base (Winnenburg et al., 2006; Urban et al., 2015; Urban et al., 2017) using BLASTP v2.2.6 with a search threshold of 1e-5 and minimum coverage of 25%. The updated fungal version of the antiSMASH v5 (antibiotics and Secondary Metabolite Analysis Shell) webserver^5^ was used to predict secondary metabolite (SM) biosynthesis gene clusters (Blin et al., 2019a,b). Domain composition of the core biosynthesis genes was verified using the NCBI’s conserved domain database (CDD) (Marchler-Bauer et al., 2017) as well as the NRPS/PKS Analysis Website (Bachmann and Ravel, 2009).

### Secretome and Effector Prediction

The fungal proteome was filtered using a series of steps to predict secreted proteins based on recommendations by Sperschneider et al. (2015) and Jones et al. (2018). To predict proteins containing signal peptides SignalP 4.1 (SP = YES) was used (Petersen et al., 2011). Predicted signal peptides were subsequently submitted to TMHMM 2.0 (Krogh et al., 2001). Proteins with no transmembrane domains (TM = 0) as well as those with TM = 1, if located in the N-terminal signal or first 60aa (Exp AA > 18 and, or first60 > 10) were kept. TargetP 1.1 (Emanuelsson et al., 2007) was used to establish if proteins were secreted or localized to the mitochondria, chloroplast, to another or unknown location. Secreted proteins (Loc:S) and those predicted as localisation other (Loc:_, with no restriction on RC score) were retained. The PredGPI prediction server^6^ was used to identify secreted proteins that contain a GPI anchor (Pierleoni et al., 2008). Cysteine content and protein length were also calculated using in-house Python scripts.

The predicted secretome was submitted to EffectorP1 (Sperschneider et al., 2016) and EffectorP2 (Sperschneider et al., 2018a) to elucidate the effector candidate complement. For this study, a more sensitive approach, as suggested by Sperschneider et al. (2018a), was followed, retaining all predicted effectors (*P* ≥ 0.5) by both EffectorP and EffectorP2. Subcellular localization of effectors were predicted using LOCALIZER (Sperschneider et al., 2017) and ApoplastP (Sperschneider et al., 2018b).

### Lifestyle Prediction

To predict the trophic lifestyle of *N. psidii* using the CAZyme-Assisted Training And Sorting of -trophy (CATAStrophy) prediction tool (Hane et al., 2020), carbohydrate-active enzyme (CAZymes) annotations were assigned to the proteome using dbCAN (dbCAN 6.0 release downloaded in February 2018) and HMMER3. The downloaded database was converted into a HMM formatted database using hmmpress and hmmscan was run with the following parameters; –domtblout results.out.dm. The hmmscan-parser.sh (from dbCAN) was used to process the results table with *e*-value 1E-3 as a filter. Trophic lifestyle prediction was then carried out using the CATAStrophy-pipeline (Hane et al., 2020) with options -profile conda and –dbcan_version 6. The species included in the CATAStrophy training set were used to define the trophic phenotype or lifestyle (Hane et al., 2020).

## Results

### Genome Sequencing and Assembly

*De novo* assembly, followed by the scaffolding of the *N. psidii* (MA3) genome, resulted in 455 scaffolds with a total length of 38.57 Mb. The largest scaffold consisted of 2,118,495 bp (Table 1), and scaffold N50 and L50 were calculated as 933,113 bp and 14, respectively. Scaffolds > 1000 bp contributed to ∼99% of the total genome size. Completeness assessment with BUSCO v3.0.2 showed that the assembly contained 289 (99.7%) complete and one fragmented orthologous gene (0.3%).

**TABLE 1:** *Nalanthamala psidii* MA3 genome and annotation features.

Features	Value
Genome size^1^	38,577,484 bp
# contigs^1^	455
# contigs (≥1,000 bp)^1^	129
# contigs (≥5,0000 bp)^1^	75
Total length (≥5,0000 bp)^1^	38,014,136 bp
Largest contig	2,118,495 bp
GC (%)	50.42
N50	14
N75	31
L50	933,113 bp
BUSCO	99.7%
Number of predicted protein-coding genes	16 889

*^1^Based on contigs of size ≥200 bp unless otherwise noted.*

### Repeat Element Analysis

**FIGURE 1:**
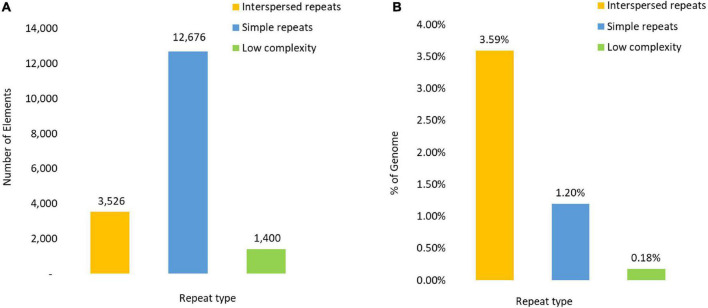
Composition of the Repetitive DNA content reflected in **(A)** as the number of repeat elements and **(B)** by the percentage of the genome covered by each repetitive element type.

Repetitive elements were found dispersed across all scaffolds covering an estimated 4.94% (2,662,768 bp) of the genome and comprising of 3,630 interspersed repeats (3.59%) as well as 12,602 simple repeats (447,835 bp) (1.18%). A total of 1,382 regions of low complexity were identified, occupying a further 65,640 bp (0.18%) of the genome (Figure 1).

Interspersed repeats were classified into two Classes; Retrotransposons (Class I) and DNA transposons (Class II). Retrotransposons was the largest class represented by 2,572 TEs, which covered 3.04% of the genome. Within the Retrotransposon class, the long terminal repeat retrotransposons (LTR) category was the largest with 1955 repeat elements distributed across five Superfamilies (Table 2), of which Gypsy and Copia were the largest represented by 372 and 183 TEs, respectively. Non-LTR retrotransposons accounted for 515 TEs. The LINE Superfamily dominated this category (Table 2), containing more than 89% of the Non-LTR TEs. Abundant LINE TE families included I (116), Jockey (115) and L1 (74), which together accounted for 58% of LINE TEs (Table 2).

**TABLE 2:** Class and Super Family composition of major families of Transposons in *N. psidii* MA3.

Class/category	Superfamily	Number of elements	Coverage (bp)	Coverage (%)
**Class I retrotransposons**	**Total:**	**2,575 (61.63%)**	**1,174,312**	**3.044%**
Class I retro-unknown/unclassified		106	9,795	0.025%
**Class I – LTR category:**				
LTR	BEL/PAO	48	2,684	0.007%
	Copia	173	17,006	0.044%
	ERV	81	5,340	0.014%
	Gypsy	345	27,149	0.070%
	Retrovirus	9	1,083	0.003%
	Unknown/unspecified	1296	1,043,421	2.705%
**Class I – non-LTR category:**				
DIRS	DIRS	14	849	0.002%
LINE	I	116	20,185	0.052%
	JOCKEY	115	11,089	0.029%
	L1	74	9,872	0.026%
	R2	12	1,023	0.003%
	RTE	16	1,434	0.004%
	Unknown/unspecified	126	20,144	0.052%
SINE	5S	2	342	0.001%
	tRNA	32	2,268	0.002%
	Unknown/unspecified	2	254	0.006%
PLE	PENELOPE	5	374	0.001%

**Class II DNA transposons**	**Total:**	**1,056 (29.08%)**	**185,270**	**0.480%**

Class II autonomous/non-autonomous/unknown		416	87,131	0.226%
**Sub-class I:**				
CRYPTON	CRYPTON	14	1,100	0.003%
TIR	CACTA	133	12,897	0.033%
	HAT	183	18,636	0.048%
	MUDR	67	13335	0.035%
	P	3	187	0.000%
	PIF-HARBINGER	32	2,578	0.007%
	PIGGYBAC	29	7,048	0.018%
	TC1-MARINER	98	36,056	0.093%
	Unknown	1	144	0.000%
**Sub-class II:**				
HELITRON		35	3,194	0.008%
MAVERICK		47	2,964	0.008%

The Class II DNA transposons were subdivided into two Sub-Classes. The Terminal Interspersed Repeat (TIR) order contained 543 TEs, while 14 TEs were classified within the Crypton order. TIR DNA transposons were contained within seven Superfamilies, of which HAT (183), EnSpm/CACTA (130), and MuDR Mutator-like (67) were the largest and contributed to 67.7% of the TIR TEs. DNA transposons of Sub-Class II were represented by two Superfamilies with 35 and 47 Helitron (Rolling circle) and Maverick (Polinton) TEs, respectively (Table 2).

### Repeat Induced Point Mutations and AT Content

**FIGURE 2:**
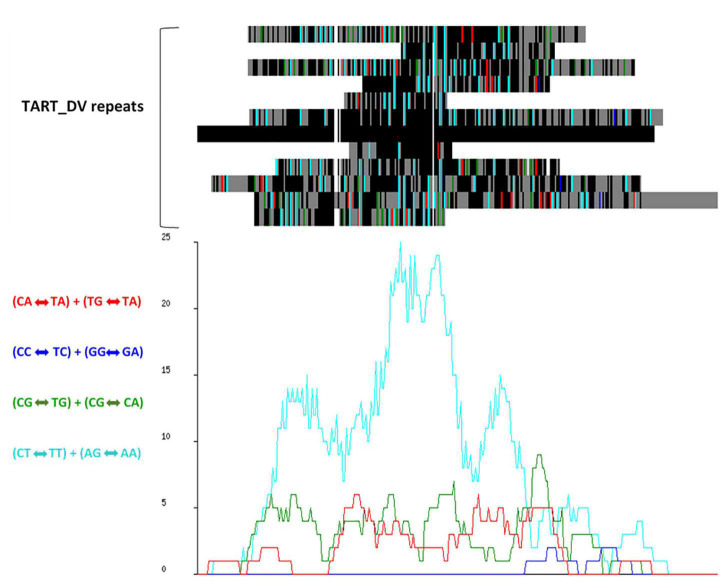
RIPCAL analysis of the TART_DV repeat family showing dominant CpT «-» TpT + ApG «-» ApA type RIP mutation. **(Top)** Multiple alignment of the putative transposon repeat family TART_DV compared to the model sequence with the highest G:C content. Black = match; gray = mismatch; white = gap. Mismatches corresponding to selected di-nucleotide changes are colored as indicated. **(Below)** RIP mutation frequency graph corresponding to the alignment above, demonstrating the prevalence of CpT «-» TpT + ApG «-» ApA mutations.

Alignment and analysis of the most prevalent repeat families showed signatures of RIP (Figure 2). The TpA/ApT index (1.5) and slightly lower (CpA + TpG)/(ApC + GpT) index (1.3), inferred from the dinucleotide frequencies within annotated repeat regions, exceeded the RIP indices threshold values that have been proposed to indicate RIP mutation in *Neurospora crassa* (Selker, 1990) of 0.89 and 1.03, respectively, providing further evidence of RIP across the genome.

**FIGURE 3:**
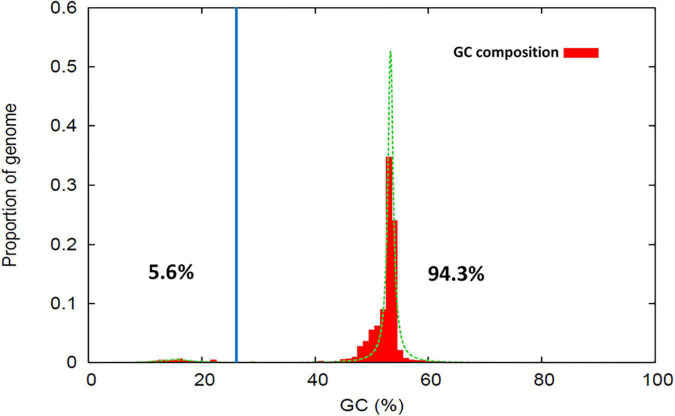
Proportion of sequence regions with distinctive GC-content profiles within the *Nalanthamala psidii* MA3 genome. The percentage values shown indicate the proportion of the genome classified within the AT-rich **(Left)** and GC-equilibrated **(Right)** peak regions.

The software Occultercut (Testa et al., 2016) was used to determine the local GC-content bias in the MA3 genome, identifying a bimodal distribution of G:C content throughout the genome. The ‘AT-rich’ peak had an average GC content of 15.5% representing only 5.66% (5.3 Kbp) of the genome and contained only 58 genes with a gene density of 26.7/Mbp. The ‘GC equilibrated’ peak had an average GC content of 53.3% and represented 94.3% of the genome. GC equilibrated regions of the genome contained 16,802 complete genes at a gene density of 464/Mbp (Figure 3).

### Functional Annotation of Protein-Coding Genes

A total of 16,889 gene models were predicted, of which 8,023 were functionally annotated, and 5,033 were assigned GO terms using InterProScan (Supplementary Table 1). Among the genes predicted, 10,771 were matched with proteins from the eggNOG fungal database, and 9,233 genes were furthermore assigned a functional description inferred from the best matching eggNOG Orthology Group. KEGG_KO and BRITE functions were assigned to 4,499 predicted genes; 2,873 of these were associated with KEGG Pathways, while 134 predicted genes were associated with BiGG metabolic reactions (Supplementary Table 2). A total of 3,425 genes showed similarity to PHI-base entries representing 156 pathogen species. The majority of these identified genes (73%) were homologs to entries from *Fusarium* spp. (32%) and *Magnaporthe* sp. (19%) (Supplementary Table 3). The majority of the identified genes (73%) were homologs from the species *Fusarium* sp. (32%), *Magnaporthe* sp. (19%), *Candida* sp. (7%), *Aspergillus* sp. (5%), Cryptococcus sp. (4%), *Botrytis* sp. (3%), *Alternaria* sp. (3%), and *Colletotrichum* sp. (2%). The highest number of homologs were found in representing genes of *F. graminearum* and *Magnaporthe oryzae.*

The trophic phenotype of *N. psidii* was predicted using the complete CAZyme gene component of the *N. psidii* proteome (Hane et al., 2020). Relative centroid distance (RCD) scores were calculated for each of the nine trophic sub-classes using CATAStrophy (Table 3). *N. psidii* was classified as broad range polymerotroph (syn. necrotroph) with an RCD score of 1 in this trophic class (Table 3).

**TABLE 3:** Summary of predicted CATAStrophy lifestyle classifications for *N. psidii*.

Lifestyle class	Mo1	Mo2	Mo3	MeE	MeI	PB	PN	*S*	*V*
RCD score^1^	0.489	0.000	0.534	0.829	0.554	**1.000**	0.791	0.613	0.155

*^1^Relative centroid distance (RCD) scores from 0 to 1 are presented for each of the nine trophic sub-classes. An RCD value of 1 (bold and underlined) indicates membership in a major trophic class and a value ≥0.95 (bold) predicts affinity for one or more trophic sub-classes. Classes include Monomertroph (syn biotroph) (Mo1); Symbiont, (Mo2); Free living yeast (Mo3); Mesotroph (syn hemibiotroph) External (MeE), Internal (MeI); Polymertroph (syn necrotroph): broad host range (PB), narrow host range (PN); saprotroph (S); vasculartroph (syn wilt, antrhacnose, rot) (V).*

### Predicted *N. psidii* Secretome

The predicted MA3 secretome comprised of 972 classically secreted proteins. Functional annotations were assigned to 713 proteins (73.3%) in the predicted secretome, and 700 (72%) were expressed *in vitro*. The remaining 259 putative proteins could not be assigned a putative function based on homology to any of the databases used in this study. The annotation results are summarized in Supplementary Table 4. A total of 256 putative secreted proteins had matching homologs in PHI-base, indicating potential roles in host-pathogen interactions.

**FIGURE 4:**
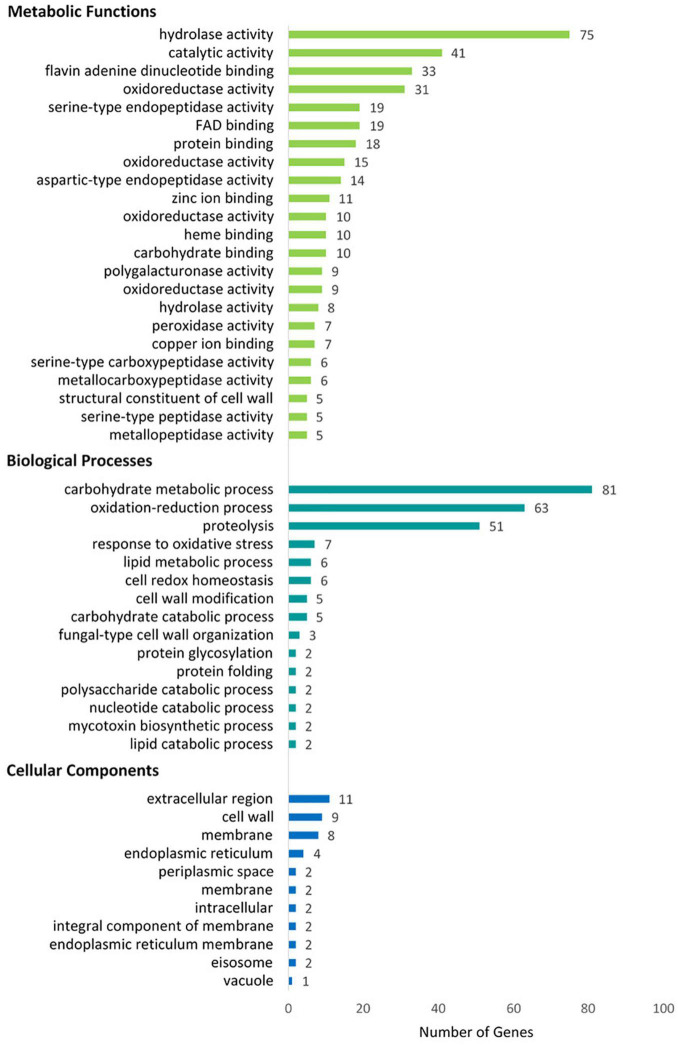
Blast2Go classification of the putative *N. psidii* MA3 secretome proteins showing top categories associated with Metabolic Functions, Biological Process, and Cellular Component categories, respectively, (COGs).

The GO terms were assigned to 365 of the secreted proteins using Blast2GO (Conesa et al., 2005; Götz et al., 2008). Sixty-two per cent (62%) of the assigned Gene Ontology (GO) terms were predicted to be involved in activities on a molecular level, 32% in Biological Processes and 6% were associated with Cellular Components (50) (Figure 4), of which 270 proteins were represented in more than one domain. Within these three domains, several over-represented functional categories were identified. These included molecular functions such as hydrolase, oxidoreductase, metal ion binding, serine-type endopeptidase, catalytic activities, and FAD- and flavin adenine dinucleotide binding (Supplementary Table 4). Within the Biological Processes, proteins associated with carbohydrate metabolic processes, proteolysis and response to oxidative stress were most abundant, while proteins associated with the extracellular region, cell wall and cell membrane were abundant in the Cellular Component category.

A total of 753 secreted proteins were assigned to eggNOG orthologous groups, of which 559 were assigned a COG category and eggNOG HMM description. The majority of the putative secreted proteins were accordingly associated with metabolism (23), while 114 were related to cellular processes and signaling, and a further 16 to information storage and processing. The most abundant functional COG categories included carbohydrate transport and metabolism (137 proteins), post-translational modification protein turnover and chaperones (77), Amino acid transport (36), as well as secondary metabolites biosynthesis, transport, and catabolism (24).

**FIGURE 5:**
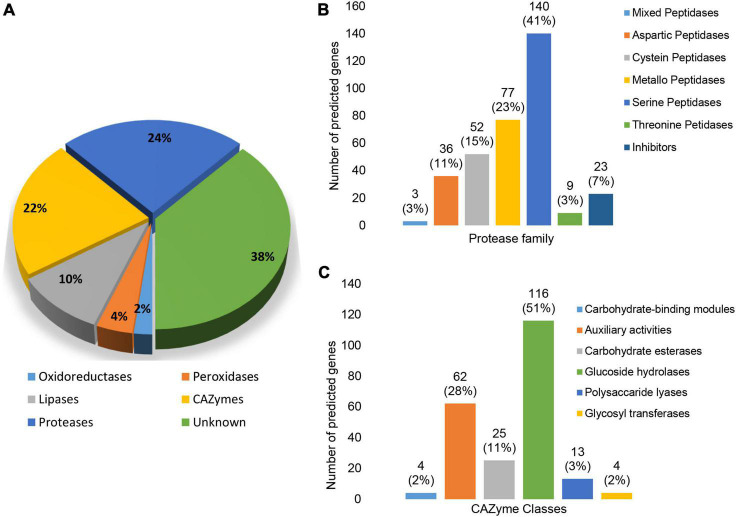
**(A)** Predicted enzyme composition of the *N. psidii* MA3 secretome. **(B)** Representation of protease families in the predicted secretome. **(C)** Representation of CAZyme families in the predicted secretome.

In addition to InterProScan and eggNOG, the results of searches against the CAZyme, Merops, and fPoxDB databases were also interrogated to further clarify the functional composition of the predicted secretome. Based on the clustering results of GO terms and COGs, secreted proteins associated with Metabolic processes featured prominently in the *N. psidii* secretome. Proteases (24%) and CAZYmes (21%) thereby contributed toward 45% of the annotated secretome with a further 10, 4, and 3% assigned to lipases, peroxidases, and oxidoreductases, respectively (Figure 5A). However, a considerable portion (38%) of the secretome remained without any functional annotation.

Twenty-four percent (24%) of the predicted secretome consisted of proteases, represented by seven protease classes (Figure 5B). Serine proteases and metallo peptidases were the most prominent classes represented by 140 (43%) putative genes. Expanded serine peptidase families included S08A (38), S09X (29), S12 (26), and S10 (12). Serine peptidase families potentially linked to plant-associated fungi (Muszewska et al., 2017), such as prolyl oligopeptidases (S9), carboxypeptidases Y (S10), D-Ala-D-Ala carboxypeptidases B (S12), prolyl aminopeptidases (S33), and sedolisins (S53) were also present in the predicted secretome. Metallo peptidases (23%) were the second most abundant protease class associated with the secretome, with families M43B (10), M28 (8), M14A (8) as most prevalent. Cysteine peptidases contributed to 15% of the proteases found in the secretome, with C15 (7), C26 (8), and C56 (9) as the most prevalent. The secretome contained a further 36 putative aspartic peptidases (10%) represented by only four families, of which aspartic peptidase family A01 with 25 proteins was the largest. The remainder of putative secreted proteases consisted of threonine peptidases (3%) and mixed peptidases (1%). A total of 23 protease inhibitors (7%) were also present, of which there were seven serine peptidase inhibitor I02 proteins and six serine carboxypeptidase Y inhibitor I51 proteins (Figure 5B).

CAZymes represented the second largest group of secreted enzymes (Figure 5C). The 224 proteins with assigned CAZyme domains were distributed across all six CAZyme classes. Glycoside hydrolases (GHs) were the most abundant contributing to 51% of secreted CAZymes followed by Auxiliary Activities (28%), carbohydrate esterases (CEs) (11%), carbohydrate binding modules (CBM) (2%), polysaccharide lyases (PLs) (6%) as well as glycosyltransferases (GTs) (2%). The most prevalent individual CAZyme classes included AA7 represented by 18 genes, GH16 by 15 genes, and GH28, GH43, CE10 and AA3 by nine, 12, 10, and 11 genes, respectively (Supplementary Table 4).

### Effectors

**FIGURE 6:**
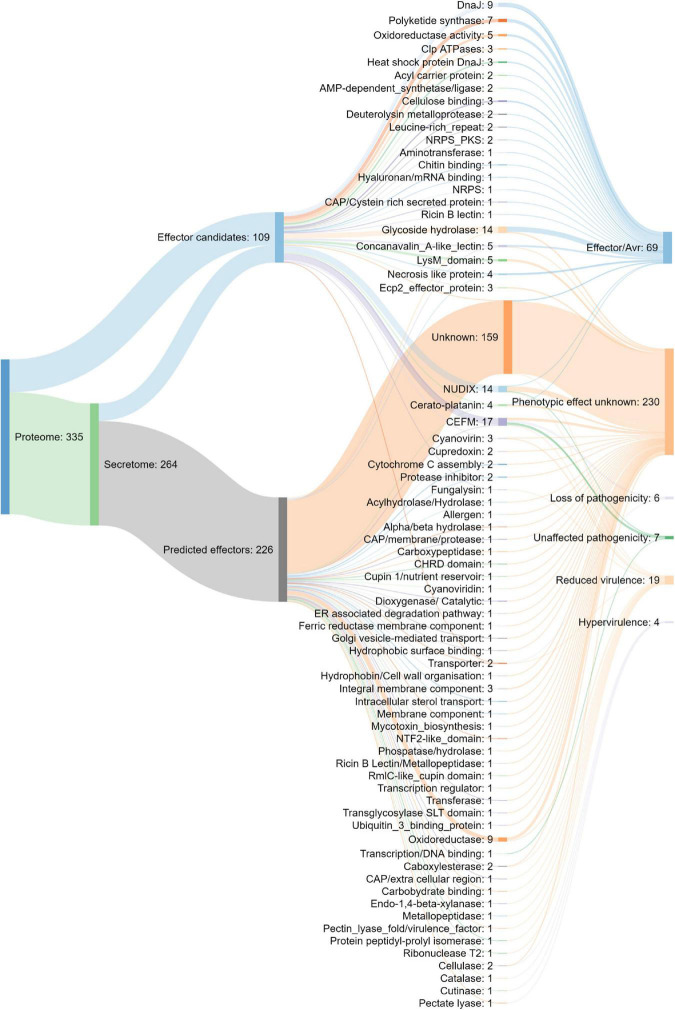
Composition of the putative *N. psidii* effecterome and predicted effect on the pathogen–host interaction of the putative PHIbase homolog.

The putative *N. psidii* MA3 effecterome was identified in three steps (Figure 6). Firstly the predicted secretome was submitted to EffectorP (Sperschneider et al., 2016) and EffectorP2 (Sperschneider et al., 2018a) and putative effectors (*p* > 0.5) identified by both predictions were then combined to allow maximum sensitivity (Sperschneider et al., 2018a). A total of 226 Effector P and P2 predicted effectors were identified, of which EffectorP predicted 197, and a further 29 additional predicted effectors were identified by Effector P2, all of which were less than 341 aa in size and contained up to 29 cysteines in their mature sequences (Figure 6). Only 68 of the Effector P and P2 predicted effectors were assigned with functional annotations (Supplementary Table 4). Secondly, in addition to the EffectorP predicted effectors a further 109 potential effector candidates were identified based on their homology to known effectors from other fungi. These effector candidates included potential pathogenicity protein homologs to LysM proteins (de Jonge et al., 2010), necrosis like proteins (NLP), Cerato-platanins (Baccelli, 2014), Cyanovirin-N lectins (Cyanovirin) (Koharudin et al., 2011), CFEM domain-containing proteins (Kulkarni et al., 2003), NUDIX hydrolases (Dong and Wang, 2016) and fungalysin (Sanz-Martín et al., 2016) (Figure 6). Lastly, the effector candidate’s ability to cause disease or trigger host response (Urban et al., 2015) was predicted through homology to PHI-base entries (Figure 6). A total of 69 potential effector candidates were homologous to plant avirulence determinants (Avrs) listed in PHIbase (Urban et al., 2017). A further four effector candidates were associated with Hypervirulence/loss of pathogenicity, six with ‘loss of pathogenicity’ (knock-out phenotype) and 19 were predicted to play a potential role in pathogen virulence (‘reduced virulence’ knock-out phenotype). Most of the effector candidates (68%) had no PHI-base homolog and their potential phenotypic effect could not be predicted (phenotypic effect unknown, Figure 6).

The localization of the cytoplasmic effectors may provide additional information on the virulence role of effectors (Sperschneider et al., 2017; Sperschneider et al., 2018b). Twenty-five effectors were predicted to localize to the apoplast, while an additional 45 effectors were predicted to target the chloroplast, mitochondria and nucleus (Supplementary Table 4).

### Secondary Metabolites

**FIGURE 7:**
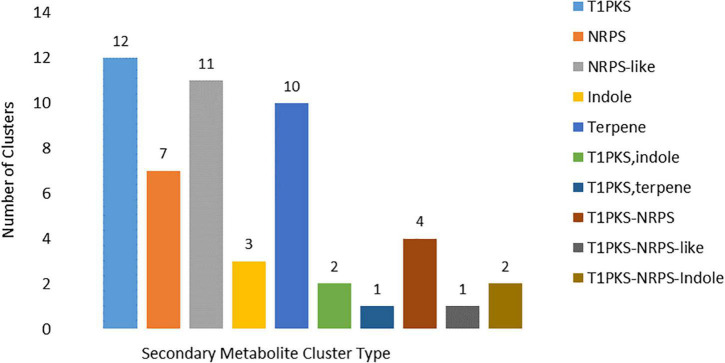
Core biosynthesis gene types associated with the identified secondary metabolite clusters types identified (T1PKS = T1 polyketide synthase; NRPS = non-ribosomal peptide synthetase).

In the MA3 *N. psidii* genome 51 putative secondary metabolite (SM) biosynthesis gene clusters (BCGs) were identified (Supplementary Table 5) using the fungal version of antiSMASH v5 webserver (Medema et al., 2011; Blin et al., 2019b). The SM-BGCs consisted of one or more core enzymes involved in the synthesis of the secondary metabolite. A total of 58 core biosynthesis genes were identified, including 12 T1 polyketide synthase (T1PKS), seven non-ribosomal peptide synthetase (NRPS), 11 NRPS-like, 10 terpene and three indole core enzymes were identified. SM-BGCs clusters with hybrid and multiple biosynthetic core genes were also present, including one T1PKS-indole and two T1PKS-NRPS hybrids, one T1PKS-NRPS-Indole, one T1PKS-NRPS-like, T1PKS-terpene and two T1PKS-NRPS multi-core combinations (Figure 7). Domain composition of the core biosynthesis genes was verified using the NCBI’s conserved domain database (CDD) (Marchler-Bauer et al., 2017) as well as the ‘NRPS/PKS Analysis Website’ (Bachmann and Ravel, 2009). Conserved domains could not be verified for the predicted core synthesis gene (MA3G_16379) of the SM BGC 62.1. Furthermore, 57 core biosynthesis genes were supported by *in vitro* expression, and 47 had PHI-base homologs. Fifteen identified SM BGCs showed similarity to known SM clusters (Supplementary Table 5). These included Macrophorin (100%), Pyriculol (23%), Duclauxin (28%), Leucinostatins (20%), Swainsonine (71%), Verticillins (76%), ACT-Toxin II (100%), Phyllostictine A/B (40%), Chaetoglobosin (28%), Botrydial (57%), Emercillin (28%), ACR-toxin (100%) as well as Nivenol/trichodiene (16%). The SM BGCs also contained several enzymes involved in the final modification of the secondary metabolite and included 146 additional biosynthetic, 33 transport-related, and five regulatory genes found, of which 233 were supported by *in vitro* expression and 122 had PHI-base homologs. An additional 671 putative ‘other’ genes formed part of the predicted BGCs of these 355 were expressed *in vitro*, and 123 had PHI-base homologs.

### Peroxidases and Oxidoreductases

Six hundred and ninety-five (695) genes were predicted to be associated with peroxidases (Supplementary Table 6), of which only 24 were considered to be secreted. Most of the genes (89%) were classified as Haem peroxidases, while only 11% were associated with non-haem peroxidases. Expanded enzyme classes included Linoleate diol synthase (PGHS like) with 332 genes, followed by haloperoxidases (45 genes), NoxA (63 genes) and Hybrid Ascorbate-Cytochrome C peroxidase with 36 genes. Eleven (11) NoxR regulators of NADH oxidases (NoxA and NoxB) were also identified (Supplementary Table 6). No Vanadium chloroperoxidase, Lignin peroxidase or Cyclooxygenases were found (Supplementary Table 6). Pfam domain searches and the RedoxiBase database (Fawal et al., 2013) were used to elucidate other oxidoreductases whereby 158 putative genes were associated with oxidoreductases other than A1 auxiliary CAZymes. Dehydroascorbate reductase and Thioredoxin families were most abundant with 45 and 44 genes, respectively, and two genes were found associated with the Ferritin-like domain family (Supplementary Table 6).

### Kinases

**FIGURE 8:**
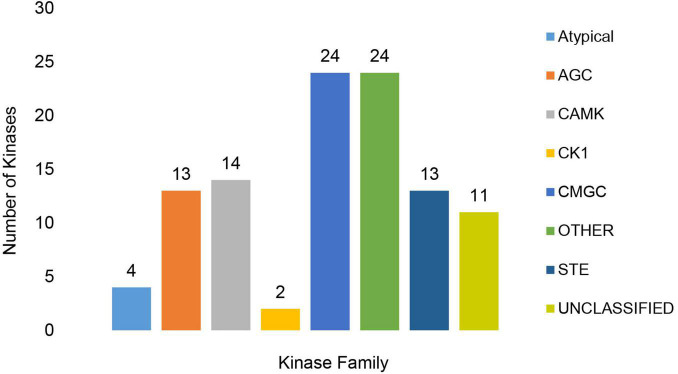
Representation of Classified and Unclassified kinase families found in the MA3 genome. The families included the Protein kinase A, G and C families (AGC), Calcium and Calmodulin-regulated kinases (CAMK), Cell Kinase 1 (CK1), CMGC group (named after the initials of some members) including key kinases such as the MAPK growth- and stress-response kinases, the cell cycle CDK (cyclin dependent kinases) as well as kinases involved in splicing and metabolic control and STE (Yeast STE homologs).

The MA3 kinome consisted of 129 Classified and Unclassified putative kinase genes encoding kinases from the major kinase families (Supplementary Table 7.1). These included 13 kinases from the protein kinase A, G and C (AGC) families, 14 Calcium and Calmodulin-regulated kinases (CAMK) and two Cell kinase-1 (CK1) kinases. The CMGC kinases group (named after the initials of some members) was represented by 24 kinases. This group includes the MAPK growth- and stress-response kinases, the cell cycle CDK (cyclin-dependent kinases), and kinases involved in splicing and metabolic control. The yeast STE homolog (STE) group contained an additional 13 kinases, while 24 ‘Other’ kinases (Figure 8 and Supplementary Tables 7.1, 7.2) were also found. Only four atypical kinases (aPKs) and no aPK/HisK kinases were detected. Furthermore, 23 UNCLASSIFIED eukaryotic protein kinase (ePKs) were present, which may be species-specific, or members of novel families. Slight expansions in the number of CAMK and CMGC PKs were detected in comparison to the ‘core’ Ascomycetes/Fusaria kinome described by DeIulio (DeIulio et al., 2018). All PKs identified were expressed *in vitro*, and 105 had PHI-base homologs, which were considered to affect virulence (Supplementary Tables 7.1, 7.2).

### Transcription Factors

A total of 643 putative transcription factors (TFs) were identified, of which 183 contained more than one TF DNA binding domain (TF DBD) motif. These dual-specific TFs, consisting of various combinations of TF families, are common in fungal species, according to Shelest (2017). Identified TF DBDs were compared to TFs typically found in fungi as described by Shelest (2017). A total of 785 TT DBDs correlated with the listed fungal TF-type DBDs. Zinc finger transcription factors (370) were the most abundant of the TF families represented. The Zinc finger, along with the Fungal Transcription factors (115), Winged helix repressors DNA-binding (55), Nucleic acid-binding OB-folds (52), Homeodomain like (IPR009057) and basic-leucine zipper (bZIP) transcription factors accounted for 82% of the total number of TFs identified (Supplementary Table 8.1). Phi-base homologs were assigned to 548 of the 643 putative TF genes and 629 were expressed *in vitro* (Supplementary Tables 8.1, 8.2).

### Cytochrome P450

**FIGURE 9:**
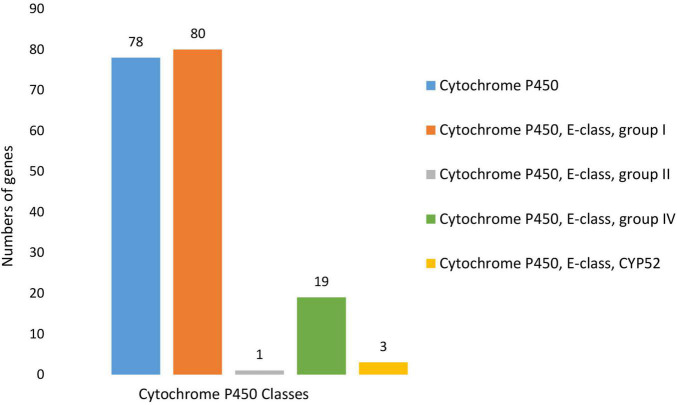
Number and distribution of putative Cytochrome P450 genes identified within the *N. psidii* genome. Predicted genes were classified according to motif type, which included Cytochrome P450, Cytochrome P450, E-class, group I, Cytochrome P450, E-class, group II, Cytochrome P450, E-class, group IV and Cytochrome P450, E-class, CYP52.

BLASTP (v 2.2.6) search against the Fungal Cytochrome P450 Database (Park et al., 2008) and further filtering carried out using InterProScan Pfam, and PRINTS resulted in the identification of 124 putative Cytochrome P450 (CYP) genes (Supplementary Table 9). The CYP genes were distributed across five CYP families, with most of the putative genes containing more than one CYP motif. Cytochrome P450 and Cytochrome P450 E-class group I motifs were present in, respectively, 78 and 80 of the identified genes. In addition to these CYP families, a further 19 genes contained Cytochrome P450, E-class, group IV motifs, three contained Cytochrome P450, E-class, CYP52 motifs, and one contained a Cytochrome P450, E-class, group II motif (Figure 9). Of the putative CYP genes identified, 109 had Phi-base homologs, and 178 were expressed *in vitro*.

### Transport Proteins

**FIGURE 10:**
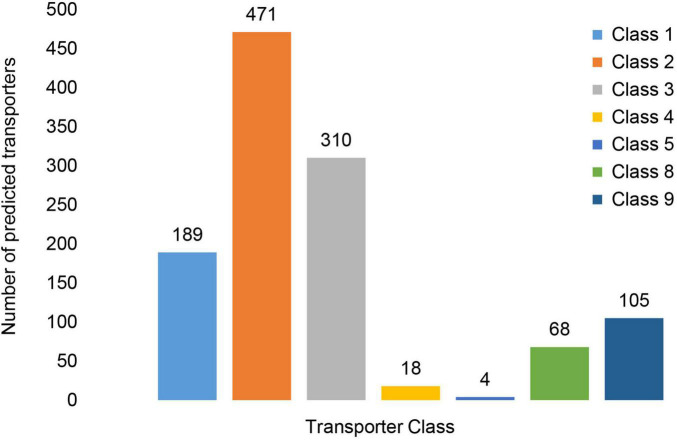
Distribution of putative transporter genes across Transporter Classes. The transporter classes present included Class1: Channels/Pores, Class 2: Electrochemical Potential-driven Transporters, Class 3: Primary Active Transporters, Class 4: Group Translocators, Class 8: Accessory Factors Involved in Transport and Class 9: Incompletely Characterized Transport Systems.

In MA3 1,280 putative transporters were identified, which accounted for approximately 11% of its proteome. More than 97% (1,248) of the transporters were expressed *in vitro*. The putative transporters identified were classified into seven transporter classes (Figure 10) represented by 21 sub-classes and a further 557 families (Supplementary Table 10). The Electrochemical Potential-driven Transporters (Class 2) were the most abundant with 471 genes. Within this class, 469 genes were associated with the Major Facilitator Superfamily (2.A.1). Primary Active Transporters (Class 3) represented by 310 genes were the second most abundant group. The important ATP-binding Cassette (ABC) Transporter Superfamily was the largest Superfamily within this Class represented by 53 genes, of which 51 were associated with drug resistance and efflux (Table 4 and Supplementary Table 10). The Class 1 Channel and Pore Transporters was the third-largest class and contained 108 predicted genes. The remaining transporter classes identified included the Group Translocators (Class 4), Transmembrane Electron Carriers (Class 5), Accessory Factors Involved in Transport (Class 8) and Incompletely Characterized Transport Systems (Class 9), which accounted for 18, four, 68 and 105 genes, respectively.

**TABLE 4:** ATP-binding cassette (ABC) transporters associated with drug efflux and resistance.

Drug resistance/transporter/exporter family	Transporters	Number of genes
Drug exporter-2 (DrugE2) Family (3.A.1.117)	3.A.1.117.3	1
(Putative) drug resistance ATPase-2 Family (3.A.1.121)	3.A.1.121.4	2
Multidrug resistance exporter (MDR) Family (ABCB) (3.A.1.201)	3.A.1.201.1	2
	3.A.1.201.3	1
	3.A.1.201.5	1
	3.A.1.201.10	3
	3.A.1.201.11	2
	3.A.1.201.16	1
	3.A.1.201.18	4
	3.A.1.201.23	1
Eye pigment precursor transporter Family (ABCG) (3.A.1.204)	3.A.1.204.9	2
Pleiotropic drug resistance Family (ABCG) (3.A.1.205)	3.A.1.205.5	1
	3.A.1.205.6	1
	3.A.1.205.7	2
	3.A.1.205.11	7
	3.A.1.205.12	2
	3.A.1.205.19	2
Drug conjugate transporter Family (ABCC) (3.A.1.208)	3.A.1.208.2	2
	3.A.1.208.8	2
	3.A.1.208.11	1
	3.A.1.208.12	2
	3.A.1.208.14	1
	3.A.1.208.27	1
	3.A.1.208.28	1
	3.A.1.208.35	1

### Identification of Mating-Type Genes

**FIGURE 11:**
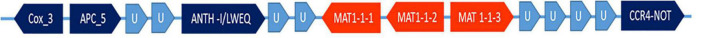
Structure of the MA3 mating type locus and flanking regions. The mating type genes MAT-1-1, MAT 1-1-2 and MAT 1-1-3 were preceded by a putative cyclooxygenase 3 (COX3) gene (MA3T_15397), Anaphase-promoting complex subunit 5 (APC_5), ANTH/LWEQ gene containing a AP180 N-terminal homology domain containing gene (ANTH) and I/LWEQ domain homologous to SLA2. Putative MA3 specific genes with no known homologs (U) were found either side of the mating type genes.

The *N. psidii* MA3 genome contained conserved Mating-type genes. Conserved mating-type genes included the MAT-α HMG-box indicative of the *MAT1-1* idiomorph (N_psidii_ MAT3_15404), *MAT1-1-2/MatA-2/Smr1* (N_psidii_MA3T_ 15405), and *MAT1-1-3* (N_psidii_MA3T_15406) in succession on MA3 Scaffold_49. The MA3 *MAT1-1-3* gene contained a MatA High Mobility Group (HMG) domain. *MAT1-2* genes were not present in the *N. psidii* MA3 genome (Figure 11). The *MAT-1-1* genes were preceded by a putative cyclooxygenase-3 (COX3) gene (MA3T_15397), Ana-phase-promoting complex subunit-5 (MA3T_15398), a gene (MA3T_15401) containing an AP180 N-terminal homology (ANTH) domain and I/LWEQ domain homologous to SLA2 as well as four possibly MA3 specific genes with no known homologs (Figure 11). In addition to the Mating type proteins, two GPCR fungal pheromone mating factor receptors, STE2 (N_psidii_MA3T_00504) and STE3 (N_psidii_MA3T_14190), as well as a mating-type switching protein (swi10), were also discovered. *In vitro* expression of both the STE and mating-type switching, proteins were detected.

## Discussion

### Secreted Enzymes and Effectors Involved in Host Colonization and the Acquisition of Nutrients

The genome of the guava wilt pathogen *N. psidii* was sequenced and assembled to obtain insight into putative genes associated with pathogenicity mechanisms. The 38.57 Mb *N. psidii* draft genome is the first reported sequenced genome for the genus *Nalanthamala*. The genome of the *N. psidii* isolate MA3 was comparable in size to reported genomes within the family of Nectriaceae such as *Geosmithia morbida* 26.5 Mb (Schuelke et al., 2016), *F. graminearum* PH-1 36 Mb (Cuomo et al., 2007), *Fusarium oxysporum* f. sp. *lycopersici* 61 Mb, (Ma et al., 2010) and *N. haematococca* 54.43 Mb (Coleman et al., 2009). A total of 16,889 protein-coding genes were identified based on *de novo* predictions and homology-based searches, which was consistent with other members within the Nectriaceae, i.e., *F. oxysporum* strain 4287 (17,735), *F. verticillioides* (14,179), and *N. haematococca* (15,707) (Coleman et al., 2009; Ma et al., 2010).

Host invasion and colonization by phytopathogens are primarily accomplished through the secretion of extracellular proteins and effectors. Therefore, identifying these secreted proteins with hypothesized functions in host penetration, tissue necrosis, immune subversion, and fungicide resistance is of great significance in understanding fungal pathogenicity and disease management (Zeng et al., 2018). The predicted *N. psidii* MA3 secretome consisted of 972 genes representing 5.8% of the complete proteome. The *N. psidii* secretome was slightly larger than the predicted secretome of *F. graminearum* (4.2%) (Brown et al., 2012) but similar in size to the necrotrophs *Botrytis cinerea* (5.6%) and *Sclerotinia sclerotiorum* (5.5%) (Amselem et al., 2011).

*Nalanthamala psidii* MA3 encoded for 671 CAZymes, of which 216 were secreted. Secreted CAZymes are essential during the infection and decomposition of the host plant tissue (Gibson et al., 2011; Zhao et al., 2013) and promote the utilization of mono- and oligosaccharides for growth and reproduction (Kubicek et al., 2014). Putative AA3 enzymes, often found in abundance in wood-degrading fungi, were prevalent in the *N. psidii* secretome. These enzymes play an important role in lignocellulose degradation, usually cooperating with other AA-enzymes such as peroxidases AA2 and glucose-methanol-choline oxidoreductase AA8. These auxiliary enzymes as well as the lytic polysaccharide monooxygenases (LMPO) AA9 and fungal LMPO AA11 involved in the oxidation of chitin, were all present in the predicted secretome (Daniel et al., 1994; Langston et al., 2011; Kracher et al., 2016). Also present in the *N. psidii* MA3 CAZyme repertoire were important pectate lyases such as PL1 and PL3 and rhamnogalacturonan endolyase PL4 responsible for the degradation of the complex pectin plant cell wall component (Collmer and Keen, 1986). These pectic enzymes are suggested to be used in the maceration of plant tissue, cell lysis and alteration of the cell wall structure, providing other depolymerising enzymes with the opportunity to exploit their respective substrates (Collmer and Keen, 1986). PL was found to play an important role in the virulence of pathogenic fungi such as *Colletotrichum coccodes* (Ben-Daniel et al., 2012) and *B. cinerea* (ten Have et al., 1998; Kars et al., 2005). Yang and Chung (2012) thereby suggested that pectate lyases could have diverse functions in pathogenesis and may provide *V. dahliae* with the ability to cause disease in a broad range of hosts. In addition, CMB50 and CMB18, which both have chitin-binding functions, were also discovered. These putative CAZymes may play a role in preventing the elicitation of host defenses and PTI (de Jonge et al., 2010). The LysM domain-containing CMB50 proteins are furthermore known fungal effectors, which were shown to inhibit plant chitinases (Marshall et al., 2011).

Necrotrophic and hemibiotrophic pathogens often secrete extensive repertoires of CAZymes, the profiles of which can be used as indicators of the fungal lifestyle (Knogge, 1996; Gibson et al., 2011; Hane et al., 2020). *N. psidii* was predicted, using the CATAStrophy lifestyle prediction tool, as a broad host range polymerotroph/necrotroph (Hane et al., 2020). Grech (2015) found that *N. psidii* was also able to induce symptoms in closely related Myrtaceae such as *Myrciaria cauliflora* (Jaboticaba), *Eugenia pseudopsidium* (Christmas cherry), and *Psidium montanum* (Mountain or Spice guava) upon inoculation, thereby confirming the suggested broader host range.

Roughly 30% (5,070 genes) of the MA3 proteome was associated with functional proteases and protease inhibitor motifs, of which only 340 were predicted to be secreted. Serine peptidases (SPs) were the most prevalent protease class within the proteome and secretome, while non-secreted SPs are suggested to be occupied with activities such as signal peptide processing, vacuole maintenance and recycling of other peptidases, secreted proteases play an essential role in nutrient acquisition and disease development (Chandrasekaran et al., 2016; Muszewska et al., 2017). Muszewska et al. (2017) also observed expansions of SP trypsins (S01) and subtilisin peptidases (S08) associated with pathogenic fungi in accordance with our findings. The *N. psidii* MA3 secretome contained several metalloproteases, including the effector fungalysin (M36, MA3T_00078). Fungalysin secreted by the maize pathogen *F. verticillioides* was suggested to cleave the chitin-binding domain of the host’s chitinase, reducing its antifungal activity. On the other hand, *F. oxysporum* f. sp. *lycopersici* deployed a combination of a serine protease and metalloprotease (fungalysin) to remove the chitin-binding domain from two tomato chitinases (Jashni et al., 2015b). Protease inhibitors were also present in the secretome and are secreted by plant pathogens to inhibit plant proteases and promote disease development (Jashni et al., 2015a). Secreted cysteine and serine PIs were found to compromise the plant’s basal defense response (Jashni et al., 2015a,b). In addition to the CAZymes and proteases, the *N. psidii* MA3 secretome contained a substantial number of lipases, including four cutinases. Cutinases have been implicated in various pathogenicity roles during plant infection, including spore attachment, degradation of the waxy plant cuticle, and nutrient acquisition (Skamnioti et al., 2008; Liu et al., 2016), while lipases, according to Subramoni et al. (2010) may be involved in the degradation of stored lipids and/or signaling through the release of secondary messengers.

Fungal phytopathogens secrete small proteins or effectors specifically targeted at manipulating host responses during infection (Varden et al., 2017). A total of 335 potential effector candidates were identified, of which 79 were further predicted to promote the pathogen’s ability to cause disease. Putative effector candidates included CFEM domain-containing proteins with possible roles in appressoria development, stress resistance and virulence (Kou et al., 2017; Zhu et al., 2017), LysM effectors which protect fungal hyphae against hydrolysis by host chitinases (de Jonge et al., 2010), as well as Cyanovirin-N lectins (Koharudin et al., 2011), known to play a role during fungus–plant interactions. As a putative necrotroph, it is likely for *N. psidii* to actively induce a hypersensitive response (HR) and cell death (Govrin and Levine, 2000). Several necrosis and ethylene-inducing peptides (NEP1) (Gijzen and Nürnberger, 2006; Oome et al., 2014), necrosis like proteins (NLPs), ECP2 (Laugé et al., 1997) as well as Cerato-platanin effectors (Baccelli, 2014) were thereby found in the *N. psidii* effectorome. These effectors are known for their ability to trigger tissue necrosis and cell surface-dependent immune responses in plant cells (Oome et al., 2014).

### Secondary Metabolites

In developing the GWD resistant TS-G2 guava variety, culture filtrates from the *N. psidii* MA3 stain were used to screen guava seedlings for resistance (Vos et al., 1998). Susceptible seedlings turned brown within 48 h after exposure to the culture filtrates. It is thereby speculated that toxins secreted *in vitro* might play a role in the browning of the seedlings. Phytopathogenic fungi often secrete toxins to facilitate host colonization by promoting the disruption of host cells and subsequent release of nutrients, thereby contributing to virulence or pathogenicity (Wolpert et al., 2002).

The MA3 genome encoded 51 SM BGCs, of which 15 could be linked to known SMs. Several of the known SMs are phytotoxins with possible or known roles in fungal virulence, including nivalenol (McCormick et al., 2013), solanapyrone (Hassan et al., 2013), botrydial (Colmenares et al., 2002), pyriculol (Jacob et al., 2017), ACT and ACR_II toxins (Tsuge et al., 2013). Nivalenol and its chemotypes belonging to the trichothecene family of sesquiterpene epoxides are suggested to be involved in the inhibition of eukaryotic protein synthesis. The production of trichothecenes by *F. graminearum* was found to be an important virulence factor in wheat head blight (McCormick et al., 2013). Botrydial, a sesquiterpene and non-host specific phytotoxin, is produced by the necrotrophic fungus *B. cinerea* during infection. The toxin induces an HR response resulting in chlorosis and cell collapse, subsequently promoting penetration and colonization by the fungus (Colmenares et al., 2002). ACT and ACR-II are host specific toxins originally identified from *Alternaria* pathotypes. ACT-toxin, an epoxy-decatrienoic acid, was required by *Alternaria alternata* f. sp. *citri tangerine* to infect tangerines and mandarins (Miyamoto et al., 2008). The ACT-toxin induced dysfunction of the plasma membrane resulting in electrolyte leakage and rapid cell death (Park and Ikeda, 2008). On the other hand, the polyketide ACR-II toxin, produced by *A. alternata* f. sp. *citri jambhiri*, is required to induce leaf spot on rough lemon. In contrast to the ACT-toxin, the pore-forming ACR toxins caused uncoupling of mitochondrial oxidative-phosphorylation resulting in the leakage of the cofactor NAD+ (Akimitsu et al., 1989). In addition, a delay or suppression of defense-related genes was also observed in the rough lemon host (Gomi et al., 2003). Usually, *A. alternata* only contains one of the (two) toxins; however, a strain pathogenic to both hosts was found to contain both ACT-toxin and ACR-toxin (Masunaka et al., 2005) similar to *N. psidii* MA3.

Secondary metabolites can also include siderophores, which are low molecular-weight chelating agents with a high affinity for ferric iron. The siderophore dimethyl-coprogen was encoded by (two) genes (MA3T_12762 and MA3T_12763) and expressed *in vitro* by MA3. The dimethyl-coprogen BGC showed homology to dimethyl-coprogen BGCs of several *Trichoderma* spp. These iron-chelating secondary metabolites play an important role in microbial interactions with plants and other microbes. Siderophores were furthermore found to contribute toward fungal virulence and protection from oxidative stress in *A. alternata* (Chen et al., 2013) and *Cochliobolus heterostrophus* (*Botrytis maydis*) (Oide et al., 2007).

The in-depth analysis of the *N. psidii* genome has revealed a diverse array of secreted polysaccharide-degrading enzymes, proteases, lipases, and peroxidases associated with plant cell wall degradation, reflecting its necrotrophic nature. The identification of effector candidates and the putative secondary metabolite clusters may function as key determinants in the pathogenicity of *N. psidii* and in the future prove valuable in the breeding (Vleeshouwers and Oliver, 2014) of guava varieties with resistance to *N. psidii.*

### Promotors of Pathogen Adaptation and Virulence

So far, it is evident that *N. psidii* can employ multiple strategies, including CWDEs, secreted proteases, lipases, effectors and secondary metabolites aimed at causing the death of host cells for nutritional purposes. However, to successfully infect and colonize the host, it would need to overcome the plant’s innate defenses, including the production of damaging reactive oxygen species (ROS) that may result in cell death (Lamb and Dixon, 1997). In order to cope, the pathogen either inhibits (Chi et al., 2009) or inactivates ROS through an enzyme-mediated antioxidant response (Aguirre et al., 2006). An estimated 5% of the genes in the *N. psidii* MA3 genome encoded for putative haem and non-haem peroxidases as well as oxidoreductases, excluding auxiliary CAZymes AA3 and AA8. More than 7% of the predicted enzymes associated with peroxidases and oxidoreductases were present in the predicted secretome and included antioxidant enzymes such as catalases, haem-peroxidases, glutathione, thioredoxin, glutaredoxin, peroxiredoxins and Cu/Zn superoxide dismutases. Secreted Cu/Zn superoxide dismutases (SOD) such as N_psidii_MA3_06807 is suggested to be involved in the inactivation of superoxide anions. These proteins were expressed by *S. sclerotiorum* during infection and found to play a role in virulence (Xu and Chen, 2013).

Fungi can also induce ROS and regulate intracellular levels of ROS to promote development and virulence. A total of 71 genes associated with NoxA, NoxB, and NoxC as well as 12 NoxR genes involved in the regulation of NoxA and B, were identified in *N. psidii* MA3. These NADPH oxidases (Nox) enzymes were shown to play an important role in fungal development during pathogenesis in both *M. oryzae* (Egan et al., 2007) and *B. cinerea* (Segmüller et al., 2008).

In addition to their involvement in ROS, redox enzymes may also contribute to fungal pathogenesis, including the oxidative breakdown of cellulose and hemicellulose, synthesizing toxins, and counteracting plant-derived phenolic compounds (Liang et al., 2018). Among the secreted peroxidases and oxidoreductases were several enzymes predicted to be involved in the degradation of lignin. These included glucose-methanol-choline (GMC) oxidoreductase (Sützl et al., 2019), chloroperoxidases (Ortiz-Bermúdez et al., 2003), FAD-linked oxidases (Levasseur et al., 2010) as well as multicopper oxidases with laccase activity (Janusz et al., 2017). Additionally, putative proteins containing both FAD linked oxidase, N-terminal (PF01565) and berberine/berberine bridge-like domains (PF08031) were also present. These flavin-dependent oxidoreductases are involved in the biosynthesis of isoquinoline and alkaloid biosynthesis, according to Baccile et al. (2016). In *N. psidii* these enzymes were furthermore all, also associated with predicted SM clusters.

Plant pathogenic fungi tend to contain a larger number of CYP genes (Chen et al., 2014). *N. psidii* MA3 contained 124 putative CYP genes similar in comparison to phytopathogens *Cryphonectria parasitica, F. graminearum* and *M. oryzae* and which, respectively, contained 121, 119, and 107 CYPs. CYPs play a central role in the biosynthesis of most fungal primary and secondary metabolites (Ichinose, 2014). A total of 25 CYPs were associated with identified SM clusters in the *N. psidii* MA3 genome which included a CYP52 DBDs (MA3T_11866) associated with the Chaetoglobosin SM cluster.

Transcription factors contributed toward 3.8% of the *N. psidii* MA3 gene content. These proteins play a vital role in the metabolic reprogramming and regulation of gene expression (Park and Ikeda, 2008; Shelest, 2017) in response to host and environmental changes. TFs with putative involvement in both pathogenicity and stress response were found in *N. psidii* MA3. N_psidii_MA3_00310, a putative Atf1 transcription factor, consisted of 4 TF DBDs, i.e., *TF_Aft1_HRR* (IPR021756), *bZip*, *TF_Aft1_HRA* (IPR021755), and *TF_Aft1_OSM* (IPR020956). According to Gao et al. (2008), this modular organization with separate specific regions allows the Atf1 TF to regulate multiple functions such as osmotic stress response (OSA) and meiotic recombination (HRA and HRR). Putative TF Ste12 (N_psidii_MA3_00504) was found to play an important role in invasive growth and pathogenicity in *F. graminearum* (Rispail and Di Pietro, 2009; Wong Sak Hoi and Dumas, 2010). bZIP (Kenne et al., 2018) and C6 zinc cluster protein TFs (MacPherson et al., 2006) have also been associated with the regulation of secondary metabolism. In *N. psidii* 17 TFs were associated with the predicted secondary metabolite clusters, of which most (11) were putative C6-zinc cluster proteins. In addition to these TFs, several Velvet factor orthologs associated with the all-important Velvet transcriptional complex were also present. These conserved transcription factors, first described in *Aspergillus nidulans* (Bayram et al., 2008) play a key role in the overall regulation of SM BGCs in fungi (Keller, 2019) and may also be involved in diverse functions such as sclerotia formation (Calvo et al., 2004) and sporulation (Krappmann et al., 2006). Association of the identified TFs with virulence factors in addition to DNA-binding elements and co-regulators could be of value in the development of novel crop protection strategies (Bahn, 2015).

Protein kinases are involved in every aspect of the regulation, sensory and response networks of organisms and play an essential role in the adaptability and pathogenicity of fungi (Hegedus et al., 2016). Despite the diversity in lifestyle, the general signaling pathways involved in pathogenicity have remained conserved, according to Turrà et al. (2014). The MA3 kinome contained 129 PK genes similar to the number of kinases present in the closely related Fusaria (127) and *F. oxysporum* species complex (131) (DeIulio et al., 2018). As in most fungi, *N. psidii* MA3 contained three mitogen-activated protein kinases (MAPK) with suggested functions in the regulation of infection-related morphogenesis, cell wall remodeling, high osmolality stress response and furthermore associated with virulence of several soil-borne pathogens that cause wilt disease symptoms in a variety of crops (Hamel et al., 2012). Putative MAPK (N_psidii_MA3T_13245) showed homology to the *F. graminearum* ePK Hog1, which maintains adaptive responses to hyperosmotic stress, contributes to virulence as well as resistance to phenyl pyrrole and dicarboximide fungicides (Lin and Chung, 2010). N_psidii_MA3T_0942 was homologous to the *F. oxysporum* MAPK gene Fmk 1, which upon deletion resulted in the failure to develop penetration hyphae and reduced expression of pectate lyase gene pl1 (Di Pietro et al., 2001). Two CK1 kinases were present in *N. psidii* MA3, as predicted for most Ascomycete fungi (DeIulio et al., 2018) which play a role in morphogenesis, proper septin assembly, endocytic trafficking, and glucose sensing. Among the expanded kinases were HAL kinases involved in stress response and the transport of small molecules such as amino acids and glucose across the plasma membrane (Hegedus et al., 2016).

Fungal pathogens rely extensively on transport proteins to exploit and interact with their environment. Transporter proteins facilitate the uptake of nutrients, the stabilization of the intracellular ion concentration in addition to the efflux or secretion of primary and secondary metabolites (Barabote et al., 2011). The *N. psidii* MA3 genome encoded 1,280 transporters. Class2 Electrochemical Potential-driven and Class 3 Primary Active Transporters together accounted for 58% of the total number of transporters encoded by the MA3 genome. The important and abundant ABC transporter and MFS Superfamilies resided within these two classes. ABC and MFS transporters are involved in the efflux of toxins or host-derived antimicrobial compounds, preventing intercellular accumulation, enabling the fungus to overcome host defenses during infection and providing resistance to fungicides (Del Sorbo et al., 2000). It was suggested that tolerance to antifungal compounds provided by the large number of ABC transporters might have contributed to the extended host range of *F. oxysporum* (Alghazali et al., 2017).

Furthermore, MFS transporters are versatile secondary carriers of small molecules involved in the uptake of sugars as well as the secretion of endogenously produced SMs and toxins (Perlin et al., 2014). In addition to the ABC and MFS transporters, *N. psidii* MA3 also encoded putative MDR and PDR transporters which can confer resistance to major classes of fungicides, including anilinopyrimidines, benzimidazoles, strobilurins and azoles, as well as natural toxins such as stilbenes. Transporters from the Multi Antimicrobial Extrusion (MATE) Family (2.A.66.1) associated with the reputed detoxification of xenobiotics such as Cu, Li, Al, and Ni metal ions and secondary plant metabolites were also present in the *N. psidii* MA3 genome.

Furthermore, transporters are also involved in the secretion of host and non-host selective toxins. A total of 23 transporters were associated with SM clusters in MA3. The majority of these transporters were MFS (10) and ABC (6) transporters, which included the MA3_15639 a siderophore iron transporter 1/trichothecene efflux pump_Tri12 (PF06609; 2.A.1.3.47) associated with the secretion of toxins such the host-non-selective polyketide perylene quinone toxin, cercosporin, and T-2 toxin, nivalenol and deoxynivalenol, respectively.

The role of the secretome in the establishment of disease is well recognized. However, it is likely that the fungal pathogen carefully choreographs the secretion of these molecules during infection and the establishment of disease (Vincent et al., 2020). The role of the regulatory and transport proteins discovered remains to be elucidated but will, as pointed out by Vincent et al. (2020), become a subject of imminent studies in advancing crop protection and sustainable agriculture.

### Evolutionary Potential of *N. psidii*

Sexual reproduction of both heterothallic and homothallic Ascomycetes is regulated by the mating-type (*MAT*) locus (Coppin et al., 1997). The MA3 *N. psidii MAT* locus contained 3 canonical genes *MAT1-1-1/MAT_alpha_1*_*HMG-box*, *MAT1-1-2/MatA-2/Smr1*, and *MAT1-1-3/MatA*_*HMG-box* situated on Scaffold_49. *N. psidii* could therefore be considered as a putatively heterothallic species requiring a compatible strain carrying the opposite *MAT 1-2* idiomorph for sex to occur (Seidl et al., 2009). The *MAT1-1-1* polypeptide gene product regulates various mating functions, including fungal pheromones, which permits the recognition of *a* and α heterothallic strains prior to fusion (Bobrowicz et al., 2002). These pheromones are suggested to be required for mating-type recognition and pre-meiotic recognition of nuclear identity (Debuchy, 1999). A single pheromone with similarity to the clock-controlled pheromone ccg-4 precursor of *Pochonia chlamydosporia* and mat-specific pheromone precursor encoded by the *mfm* gene of *Podospora comata* was also present (MA3T_02083). Secreted pheromones are recognized by G protein-coupled receptors (GPCRs) which reside on the cell surface (Xue et al., 2008). *N. psidii* MA3 encoded two GPCR fungal pheromone mating factor receptors, which showed significant similarity to the yeast pheromones Ste2 (α-factor) and Ste3 (a-factor).

The function of the *MAT1-1-2* gene, present in all Sordariomycetes (Coppin et al., 1997), as well as the *MAT1-1-3* gene, have not been fully elucidated as yet. *MAT1-1-2* was found to play a crucial role in the development of fruiting bodies after fertilization in the heterothallic *Podospora anserina* and homothallic *Sordaria macrospora* (Klix et al., 2010). The *MAT1-1-2* and *MAT1-1-3* genes were essential for sexual development in *F. graminearum* (Kim et al., 2012) but were of less importance in *N. crassa* as *MAT1-1-2* and *MAT1-1-3* mutants showed only a slight decrease in fertility (Klix et al., 2010). The regulatory role of the *MAT genes* can therefore not be considered conserved among filamentous ascomycetes (Kim et al., 2012). No *MAT 1-2* idiomorph was present in the *N. psidii* MA3 genome, suggesting that *N. psidii* is heterothallic and would therefore require a mating partner to complete the sexual cycle.

In addition to sexual reproduction, vegetative hyphal fusion may be a source of genetic variation in fungi such as *N. psidii*, which are considered to be predominantly asexual. However, when hyphal fusion takes place between fungal strains that differ in *het* specificity, the resulting heterokaryon is unstable and subjected to apoptosis (Kaneko et al., 2006). *Het* loci regulate heterokaryon incompatibility. A total of 51 genes with HET domains, including three HET-C domains, were found in MA3. Approximately 50–100 HET modules are present in most pathogenic fungi, which mediate vegetative incompatibility between incompatible individuals (Dal Molin et al., 2018). The ankyrin proteins orchestrate interactions between these HET proteins, while associated NACHT domains further regulate apoptosis or programmed cell death (Daskalov et al., 2012). A large cohort of 50 Ankyrin genes and eight NACHT-NTPase domains were present in the MA3.

The discovery of *het* genes along with mating-type genes suggests that *N. psidii* has a mixed reproduction system. This finding is significant as recombination is suggested to result in progeny with higher genetic diversity (Nieuwenhuis and James, 2016). Mutation for virulence can thereby be recombined into many different genetic backgrounds, thereby increasing the potential for accelerated adaptive evolution and adaptation to changing environments, including host resistance (McDonald and Linde, 2002; Pizarro et al., 2019). Identification of the *MAT* locus and *het* genes of *N. psidii* will enable further studies into the function of these genes in the fungi life cycle, virulence, and pathogenicity.

An estimated 4.94% of the *N. psidii* genome was occupied by repetitive elements, which is relatively low on the scale range of zero to 25% reported for most fungi (Castanera et al., 2016) however, in a similar range to that of closely related *N. haematococca* (5.1%) (Coleman et al., 2009). *De novo* TE prediction uncovered a diversity of TEs with an expansion of Class I Retrotransposons (74.1%) in comparison to Class II DNA transposons (25.9%). Class I TEs also dominated the genome of *Leptosphaeria maculans*, while Class II transposons were more prevalent in *F. oxysporum* (Dal Molin et al., 2018). Several high copy number transposons were also present, which can be the result of transposon expansions (Muszewska et al., 2011). As transposable elements (TEs) replicate and integrate into new positions, they have the potential to affect gene expression and function, contribute to genome size and evolution and result in the creation of novel genes and new gene neighborhoods (Möller and Stukenbrock, 2017; Muszewska et al., 2019). Furthermore, it has been shown that TEs can be involved in the evolution and regulation of genes associated with fungal pathogenicity (Raffaele and Kamoun, 2012; Seidl et al., 2015; Tan and Oliver, 2017).

Fungal genomes attempt to protect themselves from the activity and expansion of transposable elements through repeat-induced point mutations (RIP) (Muszewska et al., 2011). According to Cambareri et al. (1991) RIP only takes place during the sexual phase of the fungal life cycle resulting in the accumulation of G:C to A:T transition mutations in the DNA. With the likelihood of mating stages confirmed, evidence of RIP was also established, which affected an estimated 17.04 Mb of the 38.04 Mb *N. psidii* MA3 genome. RIP is suggested to result in the reduction of the GC-content and the development of distinctly AT-rich regions promoting the development of a “two-speed” genome in fungi (Cambareri et al., 1991). The AT-rich regions were suggested to provide dynamic compartments for effector genes evolution through activation, rearrangements and silencing (Dutheil et al., 2016). However, this may not be the rule for *N. psidii*, and even though 58 genes were associated with the AT-rich regions in *N. psidii*, none were associated with predicted effectors. *N. psidii* MA3 thereby displayed subtle bimodality with a relatively small proportion (5.6%) of the *N. psidii* MA3 genome occupied by AT-rich regions. *V. dahliae, M. oryzae, F. oxysporum*, and most *Metarhizium* species are considered predominantly asexual in nature and in accordance with *N. psidii* also contained a relatively low proportion of AT-rich regions (Testa et al., 2016). AT rich regions were also found to be less common within the surveyed Pezizomycotina necrotrophs such as *N. psidii* than hemibiotrophs (Testa et al., 2016). Features such as method of reproduction and dynamics of TE insertions play an important role in the shaping of the characteristics of the fungal pathogen genome (McDonald and Linde, 2002; Torres et al., 2021) and may have a profound impact on the host range it can infect (Gibson, 2019; Newman and Derbyshire, 2020).

## Conclusion

Genomics has afforded us a unique glimpse into the *N. psidii* genome and potential mechanisms associated with virulence and adaptation. The 38.57 Mb *N. psidii* genome is the first reported sequenced genome for the genus *Nalanthamala* and further contributes to the list of sequenced fungal genomes within the Nectriaceae family. The in-depth analysis of the *N. psidii* genome revealed a diverse array of secreted polysaccharide-degrading enzymes, proteases, lipases as well as peroxidases associated with plant cell wall degradation. Together these cell wall degrading enzymes, in addition to predicted effector candidates and putative secondary metabolites, reflect the necrotrophic nature of *N. psidii’*s. Putative transcription factors, kinases and transport proteins discovered brought to light potential mechanisms involved in the orchestration of the host-pathogen interaction. It is envisioned that the genomic resources established will contribute to future pathogen population studies, provide an opportunity to review control strategies, and assist in developing wilt resistant guava.

## Data Availability Statement

All raw sequencing and transcriptomic data were submitted to the Sequence Read Archive (SRA) under accession PRJNA789704 (ID 789704). The genome assembly and supporting information have been deposited at the UWA repository (doi: 10.26182/dbe0-mc39). The remaining data supporting the findings of this study are available within the article/Supplementary Material or from the corresponding author upon reasonable request.

## Author Contributions

AS-E and MS conceived the study. AS-E and PB performed data analysis. AS-E performed laboratory procedures and wrote the manuscript. DR, JH, DE, and JB commented on and revised the manuscript. All authors approved the final version.

## Conflict of Interest

The authors declare that the research was conducted in the absence of any commercial or financial relationships that could be construed as a potential conflict of interest.

## Publisher’s Note

All claims expressed in this article are solely those of the authors and do not necessarily represent those of their affiliated organizations, or those of the publisher, the editors and the reviewers. Any product that may be evaluated in this article, or claim that may be made by its manufacturer, is not guaranteed or endorsed by the publisher.

## Footnotes

1
http://www.repeatmasker.org/RepeatModeler/

2
https://github.com/TransDecoder

3
https://github.com/PASApipeline/PASA_Lite

4
http://peroxibase.toulouse.inra.fr

5
https://fungismash.secondarymetabolites.org

6
http://gpcr.biocomp.unibo.it/predgpi/
